# The Impact of Parafunctional Habits on Temporomandibular Disorders in Medical Students

**DOI:** 10.3390/jcm14155301

**Published:** 2025-07-27

**Authors:** Michał Zemowski, Yana Yushchenko, Aneta Wieczorek

**Affiliations:** 1 Student Research Group, Department of Prosthodontics and Orthodontics, Faculty of Medicine, Jagiellonian University Medical College, 31-155 Krakow, Poland; m.zemowski@gmail.com (M.Z.); yana.yushchenko39@gmail.com (Y.Y.); 2 Department of Prosthodontics and Orthodontics, Institute of Dentistry, Faculty of Medicine, Jagiellonian University Medical College, 31-155 Krakow, Poland

**Keywords:** temporomandibular disorders, DC/TMD, temporomandibular joint, parafunctional habits, oral behaviors, bruxism, medical students, jaw muscle tension, stress, young adults

## Abstract

**Background:** Temporomandibular disorders (TMD) are common musculoskeletal conditions affecting the temporomandibular joints, masticatory muscles, and associated structures. Their etiology is complex and multifactorial, involving anatomical, behavioral, and psychosocial contributors. Parafunctional habits such as clenching, grinding, and abnormal jaw positioning have been proposed as contributing factors, yet their individual and cumulative contributions remain unclear. This exploratory cross-sectional study aimed to evaluate the prevalence and severity of parafunctional habits and their association with TMD in medical students—a group exposed to elevated stress levels. Subjects were examined in Krakow, Poland, using the Diagnostic Criteria for Temporomandibular Disorders (DC/TMD) protocol. **Methods**: Participants completed a 21-item Oral Behavior Checklist (OBC) assessing the frequency of oral behaviors on a 0–4 scale. A self-reported total parafunction load was calculated by summing individual item scores (range: 0–84). Logistic regression was used to evaluate associations between individual and total parafunction severity scores and TMD presence. **Results:** The study included 66 individuals aged 19–30. TMD was diagnosed in 55 participants (83.3%). The most commonly reported habits were resting the chin on the hand (90.9%) and sleeping in a jaw-compressing position (86.4%). Notably, jaw tension (OR = 14.5; *p* = 0.002) and daytime clenching (OR = 4.7; *p* = 0.027) showed significant associations with TMD in the tested population. Each additional point in the total parafunction score increased TMD odds by 13.6% (*p* = 0.004). **Conclusions**: These findings suggest that parafunctional behaviors—especially those involving chronic muscle tension or abnormal mandibular positioning—may meaningfully contribute to the risk of TMD in high-stress student populations. Moreover, the cumulative burden of multiple low-intensity habits was also significantly associated with increased TMD risk. Early screening for these behaviors may support prevention strategies, particularly among young adults exposed to elevated levels of stress.

## 1. Introduction

Temporomandibular disorders (TMDs) comprise a heterogeneous group of musculoskeletal and functional conditions involving the temporomandibular joint (TMJ), masticatory muscles, and related anatomical structures. Common clinical manifestations include pain, limited mandibular mobility, joint clicking, and sensations of jaw locking or deviation during movement [1,2,3]. Accurate diagnosis remains challenging due to the non-specific and often overlapping nature of clinical symptoms [4,5].

As one of the most prevalent musculoskeletal dysfunctions in the orofacial region [6,7], TMD can significantly impair patients’ quality of life [8]. A recent meta-analysis reported a global prevalence of approximately 34%, with individual study estimates ranging from 5% to 88%, depending on diagnostic criteria and population characteristics. The highest rates were observed among individuals aged 18 to 60 years [7]. Notably, women are disproportionately affected, exhibiting both higher prevalence and greater symptom severity than men, along with more frequent episodes and less favorable recovery outcomes [9,10]. Although the relationship between oral behaviors and TMD has been widely explored, existing findings remain inconsistent and often inconclusive [6,11,12]. In particular, only a limited number of studies have addressed this issue in young adults exposed to chronic psychosocial stress, especially within Polish academic settings. Given its high prevalence and clinical complexity, a deeper insight into the various factors contributing to TMD is essential.

The etiology of TMD is multifactorial, encompassing anatomical, traumatic, systemic, behavioral, and psychosocial contributors [1,13,14]. Some studies have also explored systemic factors, such as vitamin D deficiency, which may affect musculoskeletal function and inflammatory regulation [15]. Among the various behavioral contributors, parafunctional behaviors have received particular attention due to their potential to exert excessive mechanical load on the masticatory system.

One of the key behavioral components implicated in the development and persistence of TMD symptoms is oral parafunction. These habits refer to non-functional, repetitive activities of the stomatognathic system that are unrelated to essential tasks such as chewing, swallowing, or speaking. Examples include tooth clenching, grinding, jaw bracing, resting the chin on the hand, and excessive gum chewing [1,11]. These behaviors may increase mechanical load on the TMJ and surrounding musculature, potentially resulting in microtrauma, inflammation, or chronic pain [16].

Bruxism, often discussed alongside general parafunctions, constitutes a distinct entity. It is typically defined as sustained or rhythmic clenching or grinding of the teeth, occurring during sleep (sleep bruxism) or wakefulness (awake bruxism), and is diagnosed using separate criteria [17,18]. While its etiopathogenesis may differ from that of other oral habits, bruxism can similarly contribute to excessive strain on the temporomandibular system [12].

Psychological stress is a well-recognized factor in the pathophysiology of TMD, influencing both muscular tension and the severity of parafunctional behaviors [19,20]. Stress has been shown to exacerbate behaviors such as clenching or bracing, possibly acting as a moderating or amplifying factor in the development and chronicity of TMD [20,21,22]. These mechanisms may explain the increased prevalence of TMD among populations experiencing chronic psychological strain, including individuals with anxiety or depression [23]. Taken together, stress may contribute to both the persistence of oral habits and the chronicity of TMD symptoms.

University students—particularly those in health-related academic programs—are frequently exposed to high levels of academic pressure, irregular sleep patterns, and increased cognitive and motor demands, which may predispose them to parafunctional habits and TMD. Previous studies have identified this subgroup as being at elevated risk [23,24,25], though they remain underrepresented in the existing literature.

This study aimed to assess the prevalence of TMD and oral parafunctions in young adults under elevated stress and to examine their association with TMD presence. We hypothesized that both selected individual parafunctions and the cumulative parafunctional burden would be positively associated with the occurrence of TMD in this population.

## 2. Materials and Methods

### 2.1. Ethics Approval

The study protocol was reviewed and approved by the Bioethics Committee of the Jagiellonian University (decision no. 1072.6120.16.2024) on 17 April 2024. All procedures were conducted in accordance with the Declaration of Helsinki and Good Clinical Practice (GCP) guidelines. Written informed consent was obtained from all participants prior to data collection.

### 2.2. Study Population

This study was conducted among students enrolled in medical-related programs (medicine, dentistry, and physiotherapy) in Krakow, Poland. Recruitment was carried out through convenience sampling, primarily via online announcements posted in university-affiliated student forums. All eligible individuals were invited to participate, aiming to ensure the highest possible number of participants within the study’s logistical and time constraints. However, this strategy may have introduced a degree of selection bias.

Inclusion criteria: Informed consent to participate, age between 18 and 30 years, and presence of a complete natural dentition (excluding third molars).

Exclusion criteria: Use of prosthetic restorations, previously diagnosed TMD, clinically significant malocclusion (Angle Class II and Class III), diagnosed musculoskeletal disorders (e.g., rheumatoid arthritis, degenerative joint disease, fibromyalgia), and Grade III tooth mobility (according to the Kantorowicz classification) [26,27,28].

### 2.3. Study Design

This study employed a cross-sectional observational design. Each participant underwent a standardized clinical examination and completed a structured questionnaire in accordance with Diagnostic Criteria for Temporomandibular Disorders (DC/TMD) [27,28], which includes both symptom-based screening and physical assessment components.

All clinical examinations were conducted at the Department of Prosthodontics, Jagiellonian University Medical College, by two trained and jointly calibrated examiners. Prior to data collection, both examiners underwent internal training and calibration to ensure consistency in diagnostic procedures. A subset of participants was examined jointly by both investigators to ensure procedural consistency between examiners.

All TMD diagnoses were made collaboratively by the two examiners, based on the integrated interpretation of clinical findings and questionnaire responses, in accordance with the DC/TMD Diagnostic Decision Tree [28,29]. The examiners were blinded to participants’ questionnaire responses during the clinical examination, minimizing the risk of observer bias in the diagnostic process.

### 2.4. Assessment Tools

TMD diagnosis was established based on a clinical examination and a structured questionnaire, in accordance with the DC/TMD protocol, which provides standardized and validated guidelines for both clinical and research applications and is widely recognized as the reference standard for TMD diagnosis. According to the DC/TMD, TMD are categorized into three major diagnostic groups: muscle disorders (Group I), joint disorders (Group II), and headaches attributed to TMD (Group III), with further subclassifications such as myalgia, disc displacement, and arthralgia [27,28,30].

To assess oral parafunctional behaviors, participants completed the self-report instrument Oral Behavior Checklist (OBC) [31], consisting of 21 items addressing both sleep-related and daytime oral habits (e.g., clenching, grinding, gum chewing, tongue pressing, jaw bracing, and object biting). Following the guidelines of the Scoring Manual for Self-Report Instruments, the frequency of each behavior was rated on a 5-point scale (0–4), with response options ranging from “never” to “4–7 times a week” or from “none of the time” to “all of the time”, depending on whether the behavior occurred during the day or at night [29].

A total parafunctional intensity score was calculated by summing the individual item scores (range: 0–84), providing a quantitative measure of each participant’s overall parafunctional burden. No categorical classification (e.g., low vs. high intensity) was applied, in accordance with the exploratory nature of the study.

### 2.5. Statistical Analysis

Statistical analysis was performed using R software (version 4.4.3; R Core Team, 2023) on a Windows 10 Pro 64-bit system (build 19045). No external R packages were required for the procedures conducted. Descriptive statistics were calculated for demographic variables, TMD diagnoses, and parafunctional scores. To evaluate associations between individual oral behaviors and the presence of TMD, univariate logistic regression models were applied. Each OBC item was analyzed in two forms: binary presence (yes/no) and intensity score (0–4). In addition, the total parafunctional intensity score was tested as a continuous predictor of TMD occurrence. Multivariate analysis could not be performed due to the limited sample size; the results are based solely on univariate models. Odds ratios (OR) with corresponding 95% confidence intervals (CI) were reported. A *p*-value below 0.05 was considered statistically significant. Effect sizes were interpreted according to thresholds proposed by Chinn (2000) [32], defining small (OR = 1.68), medium (OR = 3.47), and large (OR = 6.71) effects.

## 3. Results

### 3.1. Characteristics of the Study Group

A total of 66 participants met the eligibility criteria. The age range was 19 to 30 years, with a mean age of 23.44 ± 1.73 years. The sample included 41 females (62.12%) and 25 males (37.88%).

### 3.2. Prevalence of Temporomandibular Disorders

TMD was diagnosed in 55 participants (83.33%), while 11 individuals (16.67%) did not meet the diagnostic criteria. The most common subtype was a combined muscle–joint disorder (48.48%), followed by isolated joint disorders (21.21%) and isolated muscle disorders (13.64%).

### 3.3. Prevalence of Parafunctions

The most commonly reported parafunctional behaviors were leaning the head on the hand, such as cupping the cheek or resting the chin (90.91%); sleeping in a position that puts pressure on the jaw (e.g., on the stomach or side) (86.36%); chewing gum (74.24%); maintaining tooth contact outside of eating (i.e., pressing or touching teeth together) (68.18%); and clenching teeth during waking hours (66.67%).

In contrast, the least frequently reported behaviors were playing musical instruments involving the jaw or mouth (e.g., woodwind, brass, or string instruments) (4.55%), grinding teeth during waking hours (28.79%), holding the phone between the head and shoulder (36.36%), placing the tongue between the teeth (37.88%), pressing the tongue forcibly against the teeth (39.39%), and singing (39.39%).

### 3.4. Severity of Parafunctional Behaviors

The highest reported intensity was noted for sleeping in a position that puts pressure on the jaw (e.g., on the stomach or side), with 56.06% of participants indicating it occurred “all the time” and 24.24% “most of the time”.

Sleep bruxism—defined as clenching or grinding teeth when asleep—was reported as occurring “all the time” by 16.67% of participants and “most of the time” by 15.15%.

For clenching teeth during waking hours, 27.27% reported it as occurring “sometimes,” 16.67% “most of the time,” and 1.52% “all the time.”

Pressing, touching, or holding teeth together other than while eating was reported as “sometimes” by 27.27% and “most of the time” by 15.15%.

“Holding, tightening, or tensing jaw muscles without clenching or bringing teeth together” was reported as occurring “sometimes” (16.67%), “most of the time” (12.12%), and “all the time” (1.52%).

Other parafunctional habits were reported less frequently and with lower intensity overall. Detailed distributions of frequency and severity for each behavior are presented in Table 1.

### 3.5. Total Parafunction Intensity

The maximum possible score for parafunctional activity was 84, reflecting 21 items rated on a 0–4 scale. In the studied population, total scores ranged from 0 to 65. The mean total parafunctional intensity score was 22.98 ± 12.28, with a median of 22 and an interquartile range (IQR) of 15.25 to 30.00. These results indicate substantial interindividual variability in both the frequency and overall intensity of self-reported parafunctional behaviors.

### 3.6. Association Between Parafunction Presence and TMD

Certain parafunctional behaviors were significantly associated with increased odds of TMD. The strongest association was observed for “Holding the jaw in a rigid or tense position, such as to brace or protect it”, which increased the odds of TMD more than 14-fold (OR = 14.538; *p* = 0.002).

Two main groups of statistically significant parafunctions were identified. The first group included behaviors related to chronic muscle tension, such as holding the jaw in a rigid or tense position, such as to brace or protect it (OR = 14.538; *p* = 0.002); holding, tightening, or tensing jaw muscles without clenching or bringing teeth together (OR = 8.526; *p* = 0.010); pressing, touching, or holding teeth together other than while eating (i.e., contact between upper and lower teeth) (OR = 8.615; *p* = 0.004); and clenching teeth during waking hours (OR = 4.667; *p* = 0.027).

The second group comprised behaviors involving abnormal jaw positioning, including holding or jutting the jaw forward or to the side (OR = 8.966; *p* = 0.043) and sleeping in a position that puts pressure on the jaw (e.g., on the stomach or side) (OR = 5.714; *p* = 0.026).

Table 2 summarizes the univariate logistic regression results, detailing the associations between specific parafunctional behaviors and TMD. For improved clarity, odds ratios classified as large effect sizes (OR ≥ 6.71) have been highlighted in bold [32].

**Figure 1:**
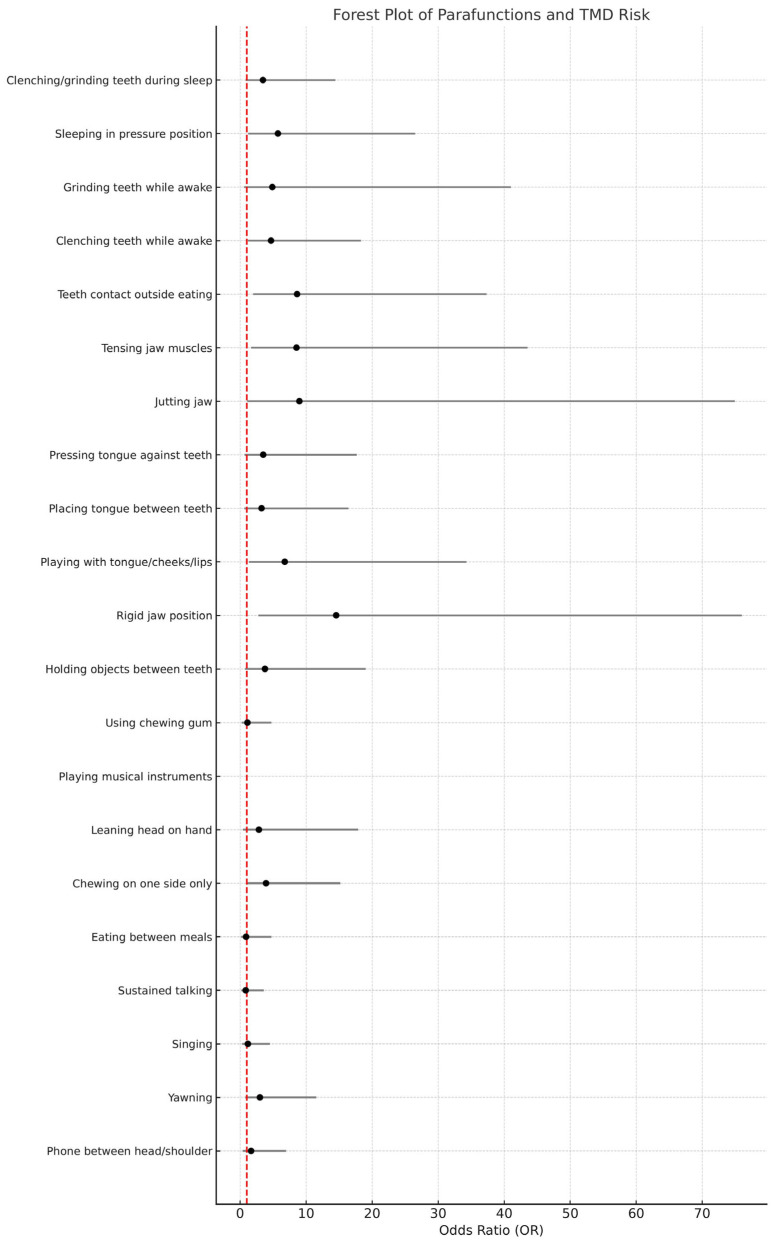
Forest plot displaying odds ratios (OR) and 95% confidence intervals for the association between individual parafunctional behaviors and TMD occurrence.

For better visual representation, the data from Table 2 were also presented as a forest plot (Figure 1), illustrating the strength and direction of associations between individual parafunctional habits and the presence of TMD.

### 3.7. Association Between Parafunction Intensity and TMD

The analysis also showed significant associations between the severity of specific parafunctions and the presence of TMD. Notably, sleeping in a position that puts pressure on the jaw (e.g., on the stomach or side) was associated with TMD at both higher frequency levels: “most of the time” (OR = 12.00; *p* = 0.044) and “all the time” (OR = 5.12; *p* = 0.048). Interestingly, even participants who reported rarely holding the jaw in a rigid or tense position, such as to brace or protect it, had significantly increased odds of TMD (OR = 7.615; *p* = 0.018). Given the exploratory nature of the study, *p*-values were not adjusted for multiple comparisons and should be interpreted with caution.

### 3.8. Association Between Total Parafunction Intensity and TMD

A significant association was observed between the total parafunction score and the presence of TMD. Logistic regression revealed that each additional point on the parafunction severity scale increased the odds of TMD by 13.6% (OR = 1.136; 95% CI: 1.042–1.238; *p* = 0.004).

## 4. Discussion

### 4.1. Main Findings

The present study aimed to evaluate the association between parafunctional habits and the occurrence of TMD in a population of medical, dental, and physiotherapy students. TMD was diagnosed in 83.3% of participants. The most frequent subtype was a combined muscle–joint disorder (48.5%), followed by isolated joint (21.2%) and muscle disorders (13.6%). These findings align with prior evidence indicating elevated TMD rates in populations exposed to chronic stress.

A wide range of parafunctional behaviors was reported, with the most common including leaning the head on the hand and sleeping in a position that puts pressure on the jaw. The analysis revealed that certain behaviors—particularly those involving chronic muscle activation (e.g., clenching, pressing the teeth together, tensing jaw muscles) and improper mandibular positioning (e.g., jutting the jaw, side-sleeping)—were significantly associated with increased odds of TMD. Notably, even infrequent engagement in certain behaviors (e.g., “rarely” holding the jaw in a rigid position) was associated with elevated risk, underscoring the potential clinical relevance of low-grade, repetitive strain.

A significant association was also found between the total parafunction intensity score and TMD, with each additional point increasing the odds of TMD by 13.6% (*p* = 0.004). In some categories, full logistic regression was not feasible due to all participants endorsing a behavior being diagnosed with TMD, potentially reflecting a strong association but also highlighting statistical limitations due to sample size.

In comparison, the study by Cannatà et al. (2025) [23], conducted among a broader university population, reported a lower prevalence of TMD: 14.0% of students presented with pain-related symptoms, 28.0% with joint-related signs, 23.4% with both, and 34.6% with neither. Notably, parafunctional behaviors were significantly more prevalent among students enrolled in science-related programs—a group to which our participants belong. These findings further underscore the need for targeted monitoring and preventive strategies in academically stressed student populations.

### 4.2. Sample Characteristics

The composition of the study sample warrants careful consideration. All participants were medical, dental, or physiotherapy students, a population known to be particularly exposed to chronic psychological stress due to academic pressure and demanding schedules [23]. While the homogeneity of the sample may limit the generalizability of our findings to the broader young adult population, it allows for focused analysis within a clearly defined subgroup. The decision to target healthcare students was intentional, as the prior literature indicates they are at elevated risk for both parafunctional behaviors and TMD symptoms [33,34].

It is important to discuss the relatively high prevalence of TMD identified in this sample (83.3%), which is elevated even for a student population. This may reflect both the elevated stress levels commonly reported among medical students and the high sensitivity of the DC/TMD criteria, which may identify mild or subclinical cases that would otherwise remain undiagnosed. Additionally, the majority of our study sample consisted of female participants, who, according to the well-established literature, are more likely to exhibit TMD symptoms [4].

### 4.3. Parafunctional Associations

The findings suggest that certain parafunctional behaviors are associated with the presence of TMD, particularly those involving chronic muscular tension and abnormal mandibular positioning. The strongest association was observed for holding the jaw in a tense or rigid position (OR = 14.538), which is consistent with previous studies highlighting sustained muscle tension as a major contributor to TMJ overload. Similarly, behaviors such as mandibular protrusion, lateral displacement, and maintaining tooth contact outside of functional mastication were also significantly associated with TMD development.

These observations support the hypothesis that not only bruxism but also other routine parafunctional activities may contribute to the etiology of functional disorders within the masticatory system [35].

A significant association was also observed between the total parafunctional score and the presence of TMD. Each additional point on the severity scale increased the odds of TMD by 13.6% (OR = 1.136; 95% CI: 1.042–1.238; *p* = 0.004), indicating that the cumulative effect of multiple mild parafunctions may play a meaningful role in TMD pathogenesis. Although no official norms have yet been established for this instrument, prior research using the OBC has indicated that a total score of 0–16 typically reflects normal behavior patterns, whereas scores of 17–24 and 25–62 occur two and seventeen times more frequently, respectively, in individuals with chronic TMD [29]. These findings reinforce the view that it is the cumulative burden of multiple parafunctions, rather than any single behavior, that may contribute to TMD development.

### 4.4. Role of Psychological Stress

An additional key consideration is the impact of psychological stress on the presence or exacerbation of TMD symptoms. Existing evidence indicates that stress may contribute to increased muscle tension and the development of unconscious parafunctional habits, such as clenching or prolonged mandibular positioning. Numerous studies have reported associations between elevated levels of stress, anxiety, or depression and a higher occurrence of TMD [18,19]. When analyzing the relationship between the Oral Behavior Checklist (OBC) score and other DC/TMD Axis II measures, Cannatà et al. found significant positive correlations between the frequency of oral behaviors and higher levels of anxiety, depressed mood, psychological distress, and somatization risk (*p* < 0.001). These findings reinforce the notion that parafunctional oral behaviors may not only reflect underlying psychological strain but also contribute to the maintenance and exacerbation of TMD symptoms.

Psychophysiological mechanisms are believed to play a central role in the etiology of TMD, particularly among young adults who are chronically exposed to emotional stress. This is especially relevant to our study population—medical students—who are recognized as being particularly vulnerable to academic and occupational stress and burnout [36].

### 4.5. Strengths and Limitations

This study has several important limitations. First, the homogeneity of the study population—comprising exclusively medical, dental, and physiotherapy students—limits the generalizability of the findings to broader young adult populations. Prior research, including the OPPERA study, has emphasized the influence of demographic variables such as age and sex on TMD prevalence, underscoring the need for more diverse samples [22]. While strict inclusion and exclusion criteria enhanced internal validity, they may have introduced selection bias by excluding individuals with more severe or atypical presentations.

Second, the study relied on self-reported parafunctional behaviors, many of which are unconscious or stress-related—such as jaw tension or clenching—which may have led to underestimation of their true frequency. Since these behaviors often occur outside of conscious awareness, participants may have inaccurately recalled or entirely overlooked them, resulting in potential misclassification. Furthermore, individuals experiencing symptoms or suspecting they had TMD may have been more motivated to participate in the study, potentially inflating the observed prevalence. Additionally, participants’ awareness of being part of a study may have influenced their responses, introducing potential bias. [37].

Third, the absence of an a priori power analysis limits the ability to definitively evaluate the adequacy of the sample size. Nonetheless, the final number of participants (N = 66) was considered sufficient to detect large effects with approximately 90% statistical power, based on meta-analytic benchmarks for TMD and masticatory muscle research [38]. However, the limited number of TMD-free participants constrained the statistical analysis. Multivariate models could not be performed, preventing adjustment for confounding factors such as age, sex, or psychological stress, and contributing to wide confidence intervals for some odds ratios. Lack of statistical significance for certain habits may also reflect limited power to detect smaller effects. Unlike large-scale prospective studies such as OPPERA [39], this analysis was restricted to univariate comparisons.

The cross-sectional design constitutes another key limitation. While associations between parafunctional behaviors and TMD were observed, causality cannot be inferred. These findings should therefore be interpreted as correlational and hypothesis-generating rather than confirmatory. Moreover, no statistical correction for multiple comparisons (e.g., Bonferroni or false discovery rate) was applied, given the exploratory nature of the study.

Although stress levels were not formally assessed, the previous literature consistently identifies elevated levels of stress, anxiety, and maladaptive coping behaviors in medical student populations [33]. These psychosocial stressors may help explain the high prevalence of TMD symptoms and parafunctional habits observed in our sample. In the absence of a standardized psychometric tool (e.g., Perceived Stress Scale), our assumption of elevated stress levels remains speculative. This limits the ability to quantify emotional distress and its contribution to the observed outcomes.

To address these limitations, future research should include larger and more heterogeneous populations to enhance generalizability and allow for multivariate modeling. Longitudinal designs would enable assessment of temporal dynamics and potential causality between parafunctional habits and TMD development. Incorporating objective behavioral assessments (e.g., surface electromyography) and validated psychometric instruments could improve the precision of measurements and support more comprehensive statistical analyses.

### 4.6. Clinical Implications

This study contributes to the growing body of evidence indicating that everyday oral behaviors—beyond classic bruxism—may play a substantial role in TMD etiology. Specifically, it identifies parafunctional habits such as prolonged jaw tension and abnormal mandibular positioning as potential contributors to TMD and emphasizes the cumulative effect of multiple low-grade oral behaviors. These insights highlight the clinical relevance of assessing behavioral factors in a medically educated, high-stress student population.

Importantly, not all individual behaviors showed statistically significant associations with TMD, possibly reflecting differences in mechanical sensitivity or psychological resilience. The broad variability in OBC scores observed in our cohort underscores considerable interindividual differences, supporting the need for further research to define diagnostic thresholds and predictive models in diverse populations. As a practical step, integrating OBC-based screening into student health programs may help identify at-risk individuals and guide targeted prevention.

From a clinical standpoint, these findings support the inclusion of behavioral and psychosocial screening into routine TMD diagnostics. Early identification of chronic jaw tension and maladaptive mandibular positioning may facilitate timely interventions. Furthermore, stress-reduction strategies and patient education on oral behaviors could become valuable components of comprehensive TMD prevention and management protocols, particularly in vulnerable populations such as students and young adults.

While the inclusion of a non-medical control group could have strengthened comparative analysis, this was beyond the scope of the present study. Future research should incorporate broader student populations to assess whether the observed associations are unique to healthcare students or generalizable across academic disciplines.

To build on these insights, future studies should aim to replicate and expand the current findings in larger, more heterogeneous cohorts. Longitudinal designs would be particularly valuable to clarify the temporal dynamics and potential causality between parafunctional habits and TMD onset or persistence. Incorporating objective behavioral assessments and validated psychometric tools would also enable more comprehensive statistical modeling and enhance the translational value of future research.

## 5. Conclusions

Despite its limitations, this study provides valuable insights into the relationship between parafunctional habits and TMDs. The results demonstrate a clear association between specific oral behaviors and the presence of TMD in young adults exposed to elevated psychosocial stress. These findings underscore the importance of early detection and routine monitoring of parafunctional habits—particularly in academic settings where chronic psychological burden is common.

Given that many of these behaviors are unconscious or subclinical, behavioral screening could serve as a useful component of early risk assessment. Clinicians should remain attentive not only to classic signs such as bruxism but also to subtle and cumulative behaviors like sustained jaw tension or habitual mandibular positioning, which may contribute to muscle overload and joint dysfunction.

Integrating behavioral assessment and targeted counseling into standard diagnostic and treatment frameworks may improve the early management of TMD, particularly in vulnerable populations. These findings provide a preliminary but compelling rationale for future longitudinal and interventional studies aimed at clarifying causality and informing preventive strategies.

## Figures and Tables

**Table 1:** Frequency and intensity of parafunctional behaviors.

Activities During Sleep	Frequency				
	Never	<1 Night /Month	1–3 Nights /Month	1–3 Nights /Week	4–7 Nights /Week
Clenching or grinding teeth when asleep	32 (48.48%)	6 (9.09%)	7 (10.61%)	10 (15.15%)	11 (16.67%)
Sleeping in a position that puts pressure on the jaw (e.g., on the stomach or side)	9 (13.64%)	2 (3.03%)	2 (3.03%)	16 (24.24%)	37 (56.06%)
Activities During Waking Hours	Frequency				
	None of the time	A little of the time	Some of the time	Most of the time	All of the time
Grinding teeth during waking hours	47 (71.21%)	12 (18.18%)	5 (7.58%)	2 (3.03%)	0 (0.00%)
Clenching teeth during waking hours	22 (33.33%)	14 (21.21%)	18 (27.27%)	11 (16.67%)	1 (1.52%)
Pressing, touching, or holding teeth together other than while eating (i.e., contact between upper and lower teeth)	21 (31.82%)	17 (25.76%)	18 (27.27%)	10 (15.15%)	0 (0.00%)
Holding, tightening, or tensing jaw muscles without clenching or bringing teeth together	28 (42.42%)	18 (27.27%)	11 (16.67%)	8 (12.12%)	1 (1.52%)
Holding or jutting the jaw forward or to the side	39 (59.09%)	17 (25.76%)	8 (12.12%)	1 (1.52%)	1 (1.52%)
Pressing the tongue forcibly against the teeth	40 (60.61%)	14 (21.21%)	7 (10.61%)	5 (7.58%)	0 (0.00%)
Placing the tongue between the teeth	41 (62.12%)	16 (24.24%)	7 (10.61%)	2 (3.03%)	0 (0.00%)
Biting, chewing, or playing with the tongue, cheeks, or lips	31 (46.97%)	11 (16.67%)	15 (22.73%)	6 (9.09%)	3 (4.55%)
Holding the jaw in a rigid or tense position, such as to brace or protect it	22 (33.33%)	24 (36.36%)	8 (12.12%)	8 (12.12%)	4 (6.06%)
Holding objects between the teeth (e.g., pipe, pencil, pens, fingers, fingernails)	39 (59.09%)	15 (22.73%)	8 (12.12%)	3 (4.55%)	1 (1.52%)
Using chewing gum	17 (25.76%)	20 (30.30%)	18 (27.27%)	9 (13.64%)	2 (3.03%)
Playing musical instruments that require use of the jaw or mouth (e.g., woodwind, brass, strings)	63 (95.45%)	2 (3.03%)	1 (1.52%)	0 (0.00%)	0 (0.00%)
Leaning the head on the hand, such as cupping or resting the chin	6 (9.09%)	11 (16.67%)	33 (50.00%)	13 (19.70%)	3 (4.55%)
Chewing food on one side only	24 (36.36%)	17 (25.76%)	14 (21.21%)	6 (9.09%)	5 (7.58%)
Eating between meals (i.e., food that requires chewing)	13 (19.70%)	19 (28.79%)	21 (31.82%)	10 (15.15%)	3 (4.55%)
Sustained talking (e.g., teaching, sales, customer service)	20 (30.30%)	23 (34.85%)	13 (19.70%)	7 (10.61%)	3 (4.55%)
Singing	40 (60.61%)	20 (30.30%)	2 (3.03%)	4 (6.06%)	0 (0.00%)
Yawning	17 (25.76%)	23 (34.85%)	19 (28.79%)	4 (6.06%)	3 (4.55%)
Holding the telephone between the head and shoulder	42 (63.64%)	19 (28.79%)	5 (7.58%)	0 (0.00%)	0 (0.00%)

Frequency and intensity of reported oral parafunctional behaviors. Values represent the number of participants and percentage (in parentheses) who selected each response option. The first two rows refer to nocturnal behaviors; the remaining items to behaviors during wakefulness.

**Table 2:** Association between the presence of parafunctions and the occurrence of TMD.

Parafunction		N	n	OR	95%CI		*p* < 0.05
Clenching or grinding teeth when asleep	No	32	24	1	ref.		
	Yes	34	31	3.444	0.824	14.392	0.09
Sleeping in a position that puts pressure on the jaw (e.g., on the stomach or side)	No	9	5	1	ref.		
	Yes	57	50	5.714	1.232	26.505	0.026 *
Grinding teeth during waking hours	No	47	37	1	ref.		
	Yes	19	18	4.865	0.577	40.992	0.146
Clenching teeth during waking hours	No	22	15	1	ref.		
	Yes	44	40	4.667	1.193	18.26	0.027 *
Pressing, touching, or holding teeth together other than while eating (i.e., contact between upper and lower teeth)	No	21	13	1	ref.		
	Yes	45	42	**8.615**	1.99	37.301	0.004 *
Holding, tightening, or tensing jaw muscles without clenching or bringing teeth together	No	28	19	1	ref.		
	Yes	38	36	**8.526**	1.671	43.509	0.01 *
Holding or jutting the jaw forward or to the side	No	39	29	1	ref.		
	Yes	27	26	**8.966**	1.073	74.894	0.043 *
Pressing the tongue forcibly against the teeth	No	40	31	1	ref.		
	Yes	26	24	3.484	0.688	17.643	0.132
Placing the tongue between the teeth	No	41	32	1	ref.		
	Yes	25	23	3.234	0.638	16.395	0.156
Biting, chewing, or playing with the tongue, cheeks, or lips	No	31	22	1	ref.		
	Yes	35	33	**6.75**	1.33	34.257	0.021 *
Holding the jaw in a rigid or tense position, such as to brace or protect it	No	22	13	1	ref.		
	Yes	44	42	**14.538**	2.782	75.967	0.002 *
Holding objects between the teeth (e.g., pipe, pencil, pens, fingers, fingernails)	No	39	30	1	ref.		
	Yes	27	25	3.75	0.741	18.978	0.11
Using chewing gum	No	17	14	1	ref.		
	Yes	49	41	1.098	0.255	4.724	0.9
Playing musical instruments that require use of the jaw or mouth (e.g., woodwind, brass, strings)	No	63	52	1	ref.		
	Yes	3	3	---	---	---	---
Leaning the head on the hand, such as cupping or resting the chin	No	6	4	1	ref.		
	Yes	60	51	2.833	0.45	17.829	0.267
Chewing food on one side only	No	24	17	1	ref.		
	Yes	42	38	3.912	1.009	15.164	0.048 *
Eating between meals (i.e., food that requires chewing)	No	13	11	1	ref.		
	Yes	53	44	0.889	0.168	4.715	0.89
Sustained talking (e.g., teaching, sales, customer service)	No	20	17	1	ref.		
	Yes	46	38	0.838	0.198	3.555	0.811
Singing	No	40	33	1	ref.		
	Yes	26	22	1.167	0.305	4.463	0.822
Yawning	No	17	12	1	ref.		
	Yes	49	43	2.986	0.775	11.499	0.112
Holding the telephone between the head and shoulder	No	42	34	1	ref.		
	Yes	24	21	1.647	0.393	6.911	0.495

*p*-values from univariate logistic regression. N—total in group; n—number of participants with TMD; OR—odds ratio; CI—confidence interval. Statistically significant associations (*p* < 0.05) are marked with an asterisk. Interpretation of effect sizes: small (OR ≥ 1.68), medium (OR ≥ 3.47), and large (OR ≥ 6.71). Large effect sizes have been highlighted in bold.

## Data Availability

The data supporting the findings of this study are available from the corresponding author upon reasonable request.

## References

[1] Chisnoiu A.M., Picos A.M., Popa S., Chisnoiu P.D., Lascu L., Picos A., Chisnoiu R. (2015). Factors involved in the etiology of temporomandibular disorders—A literature review. Clujul Med..

[2] Progiante P., Pattussi M., Lawrence H., Goya S., Grossi P., Grossi M. (2015). Prevalence of Temporomandibular Disorders in an Adult Brazilian Community Population Using the Research Diagnostic Criteria (Axes I and II) for Temporomandibular Disorders (The Maringá Study). Int. J. Prosthodont..

[3] Paulino M.R., Moreira V.G., Lemos G.A., da Silva P.L.P., Bonan P.R.F., Batista A.U.D. (2018). Prevalence of signs and symptoms of temporomandibular disorders in college preparatory students: Associations with emotional factors, parafunctional habits, and impact on quality of life. Prevalência de sinais e sintomas de disfunção temporomandibular em estudantes pré-vestibulandos: Associação de fatores emocionais, hábitos parafuncionais e impacto na qualidade de vida. Cienc. Saude Coletiva.

[4] Kapos F.P., Exposto F.G., Oyarzo J.F., Durham J. (2020). Temporomandibular disorders: A review of current concepts in aetiology, diagnosis and management. Oral Surg..

[5] Akhter R., Morita M., Ekuni D., Hassan N.M.M., Furuta M., Yamanaka R., Matsuka Y., Wilson D. (2013). Self-reported aural symptoms, headache and temporomandibular disorders in Japanese young adults. BMC Musculoskelet. Disord..

[6] Alrizqi A.H., Aleissa B.M. (2023). Prevalence of Temporomandibular Disorders Between 2015–2021: A Literature Review. Cureus.

[7] Zieliński G., Pająk-Zielińska B., Ginszt M. (2024). A Meta Analysis of the Global Prevalence of Temporomandibular Disorders. J. Clin. Med..

[8] Orzeszek S.M., Piotr S., Waliszewska-Prosół M., Jenca A., Osiewicz M., Stolarz A.P., Winocur O., Zietek M., Bombała W., Wieckiewicz M. (2023). Relationship between pain severity, satisfaction with life and the quality of sleep in Polish adults with temporomandibular disorders. Dent. Med. Probl..

[9] Haggman-Henrikson B., Liv P., Ilgunas A., Visscher C.M., Lobbezoo F., Durham J., Lövgren A. (2020). Increasing gender differences in the prevalence and chronification of orofacial pain in the population. Pain.

[10] Lövgren A., Vallin S., Häggman-Henrikson B., Kapos F.P., Peck C.C., Visscher C.M., Liv P. (2025). Women are worse off in developing and recovering from temporomandibular disorder symptoms. Sci. Rep..

[11] Šimunović L., Lapter Varga M., Negovetić Vranić D., Čuković-Bagić I., Bergman L., Meštrović S. (2024). The role of malocclusion and oral parafunctions in predicting signs and symptoms of temporomandibular disorders—A cross-sectional study. Dent. J..

[12] AlSahman L., AlBagieh H., AlSahman R. (2023). Association of stress, anxiety and depression with temporomandibular disorders in young adults—A systematic review. Arch. Med. Sci..

[13] Greene C.S. (2001). The etiology of temporomandibular disorders: Implications for treatment. J. Orofac. Pain.

[14] Ohrbach R., Dworkin S. (2016). The evolution of TMD diagnosis: Past, present, future. J. Dent. Res..

[15] Ferrillo M., Lippi L., Giudice A., Calafiore D., Paolucci T., Renò F., Migliario M., Fortunato L., Invernizzi M., de Sire A. (2022). Temporomandibular Disorders and Vitamin D Deficiency: What Is the Linkage between These Conditions? A Systematic Review. J. Clin. Med..

[16] Zieliński G., Pająk A., Wójcicki M. (2024). Global Prevalence of Sleep Bruxism and Awake Bruxism in Pediatric and Adult Populations: A Systematic Review and Meta-Analysis. J. Clin. Med..

[17] Zieliński G., Pająk-Zielińska B., Pająk A., Wójcicki M., Litko-Rola M., Ginszt M. (2025). Global co-occurrence of bruxism and temporomandibular disorders: A meta-regression analysis. Dent. Med. Probl..

[18] Mortazavi N., Tabatabaei A.H., Mohammadi M., Rajabi A. (2023). Is bruxism associated with temporomandibular joint disorders? Asystematic review and meta-analysis. Evid. Based Dent..

[19] Wieckiewicz M., Paradowska-Stolarz A., Wieckiewicz W. (2014). Psychosocial aspects of bruxism: The most paramount factor influencing teeth grinding. Biomed Res. Int..

[20] Pavlou I.A., Spandidos D.A., Zoumpourlis V., Papakosta V.K. (2024). Neurobiology of bruxism: The impact of stress (Review). Biomed. Rep..

[21] Jathanna R.V., Bangera R., Naik M.K., Jathanna V.R., Adhikari S., Vats S. (2024). Unraveling the Relationship between Oral Habits and Anxiety: A Narrative Review. J. Pharm. Bioallied Sci..

[22] Pereira L.J., Pereira-Cenci T., Pereira S.M., Cury A.A.D.B., Ambrosano G.M.B., Pereira A.C., Gavião M.B.D. (2009). Psychological factors and the incidence of temporomandibular disorders in early adolescence. Braz. Oral. Res..

[23] Cannatà D., Galdi M., Caggiano M., Acerra A., Amato M., Martina S. (2025). Prevalence of Signs and Symptoms of Temporomandibular Disorders and Their Association with Emotional Factors and Waking-State Oral Behaviors on University Students: ACrossSectional Study. Healthcare.

[24] Alam M.K., Al Shayeb M., Natarajan P.M., Abutayyem H., Di Blasio M., Marrapodi M.M., Cicciù M., Minervini G. (2025). Association of temporomandibular disorders and other jaw anomalies in chewing gum users—A systematic review. J. Oral. Facial Pain. Headache.

[25] Angeles-García K., Ladera-Castañeda M., Castro-Ramirez L., Paucar-Rodríguez E., Castro-Rojas M., Cervantes-Ganoza L., Cayo-Rojas C. (2025). Presence of TMD-related pain and symptoms associated with anxiety in Peruvian students in their final years of dental education: An analytical cross-sectional study under a multivariable regression model. BMC Oral. Health.

[26] Dejak B. (2020). Vademecum Wykonywania Protez Stałych i Ruchomych.

[27] Osiewicz M., Ciapała B., Bolt K., Kołodziej P., Więckiewicz M., Ohrbach R. (2024). Diagnostic Criteria for Temporomandibular Disorders (DC/TMD): Polish Assessment Instruments. Dent. Med. Probl..

[28] Schiffman E., Ohrbach R., Truelove E., Look J., Anderson G., Goulet J.-P., List T., Svensson P., Gonzalez Y., Lobbezoo F. (2014). Diagnostic Criteria for Temporomandibular Disorders (DC/TMD) for Clinical and Research Applications: Recommendations of the International RDC/TMD Consortium Network and Orofacial Pain Special Interest Group. J. Oral Facial Pain Headache.

[29] Ohrbach R., Knibbe W. Diagnostic Criteria for Temporomandibular Disorders: Scoring Manual for Self-Report Instruments. Version 29 May 2016. https://www.rdc-tmdinternational.org.

[30] Ohrbach R., List T., Goulet J.-P., Svensson P. (2010). Recommendations from the International Consensus Workshop: Convergence on an orofacial pain taxonomy. J. Oral Rehabil..

[31] Markiewicz M.R., Ohrbach R., McCall W.D. (2006). Oral behaviors checklist: Reliability of performance in targeted waking-state behaviors. J. Orofac. Pain.

[32] Chinn S. (2000). A simple method for converting an odds ratio to effect size for use in meta analysis. Stat. Med..

[33] Wang J., Liu M., Bai J., Chen Y., Xia J., Liang B., Wei R., Lin J., Wu J., Xiong P. (2023). Prevalence of common mental disorders among medical students in China: A systematic review and meta-analysis. Front. Public Health.

[34] Vo T.N.M., Chiu H.-Y., Chuang Y.-H., Huang H.-C. (2023). Prevalence of Stress and Anxiety Among Nursing Students: A Systematic Review and Meta-analysis. Nurse Educ..

[35] Boening K., Wieckiewicz M., Paradowska-Stolarz A., Wiland P., Shiau Y.-Y. (2015). Temporomandibular disorders and oral parafunctions: Mechanism, diagnostics, and therapy. BioMed Res. Int..

[36] MacAulay R., Morash J., Kenwell L.S., Haslam S.K. (2023). Burnout in oral health students: A scoping review. J. Dent. Educ..

[37] Gachabayov M., Dyatlov A., Bergamaschi R. (2019). Hawthorne Effect Should Be Controlled for in Quality Control Studies. JAMA Surg..

[38] Zieliński G., Gawda P. (2025). Defining Effect Size Standards in Temporomandibular Joint and Masticatory Muscle Research. Med. Sci. Monit..

[39] Slade G.D., Bair E., By K., Mulkey F., Baraian C., Rothwell R., Reynolds M., Miller V., Gonzalez Y., Gordon S. (2011). Study methods, recruitment, sociodemographic findings, and demographic representativeness in the OPPERA study. J. Pain.

